# The Relationship between Negative Leadership Behaviours and Silence among Nurses

**DOI:** 10.1155/2024/4561005

**Published:** 2024-08-06

**Authors:** Zhi-Ying Li, Yu-Pei Yang, Qian Wang, Tao-Hsin Tung, Hai-Xiao Chen

**Affiliations:** ^1^ Taizhou Hospital of Zhejiang Province Affiliated to Wenzhou Medical University, Linhai 317000, Zhejiang, China; ^2^ Department of Hematology Taizhou Hospital of Zhejiang Province Affiliated to Wenzhou Medical University, Linhai 317000, Zhejiang, China; ^3^ Evidence-Based Medicine Center Taizhou Hospital of Zhejiang Province Affiliated to Wenzhou Medical University, Linhai 317000, Zhejiang, China; ^4^ Taizhou Hospital of Zhejiang Province Zhejiang University, Linhai, Zhejiang, China

## Abstract

*Background*: Negative leadership behaviour is very common and pervasive in nursing and healthcare, often leading to adverse effects such as nurses' silence, decreased job performance, and turnover. However, there is a lack of systematic reviews that summarize negative leadership types and nurses silencing behaviours. *Aim*: This systematic review examined the relationship between negative leadership behaviour and nurses' silence. *Evaluation*: We searched PubMed, Embase, the Cochrane Library, Web of Science, CNKI, VIP, and Wanfang databases from their inception until 30 April 2024 for articles examining the relationship between negative leadership and silent behaviour among nurses. The studies were reviewed in accordance with the Preferred Reporting Items for Systematic Reviews and Meta-Analyses (PRISMA) guidelines. The risk of bias in the included studies was assessed using the Newcastle–Ottawa Scale. *Key Issues*: After full-text analysis, six papers were included in this systematic review. Four of the studies were conducted with nurses, and the remaining two were conducted with all medical staff including nurses. While most studies have shown that negative leadership causes nurses to become increasingly silent, others have shown that appropriate negative leadership behaviour instead reduces nurses' silence. *Conclusions*: Negative leadership behaviour can affect the silent behaviour of nurses. *Implications for Nursing Management*: Hospitals need to take the initiative to build a harmonious and safe working environment, correctly recognize and identify negative management behaviour, take appropriate and effective measures to enhance the positive leadership of nurse managers, and make an effort to prevent nursing staff's exposure to the negative management of direct leadership and mental health threats, which is a key point that hospital administrators and health policymakers tend to overlook. This is also effective for enhancing the leadership of hospital administrators.

## 1. Introduction

Leadership behaviour affects employees' work behaviour, organisational goals, and efficiency and has been a continual interest in management research. Most studies have focused on positive leadership behaviours and their positive effects, whereas little attention has been paid to the effects of negative leadership behaviours on employees and organisations [1]. Negative leadership behaviour refers to behaviour that leads to potential physical, psychological, emotional, or economic damage to subordinates due to the abuse of power in the process of managing employees [1]. Abusive supervision, defined as “verbal and nonverbal behaviours perceived by subordinates as hostile by their superiors, excluding physical contact behaviours,” is the most common manifestation of negative leadership behaviour [2]. In addition, authoritative leadership and toxic leadership are also typically negative leadership behaviours. Authoritative leaders are those who emphasise that their power is absolute and unchallengeable [3]. Hoffman and Sergio described disregarding employees' wellbeing and participating in actions and activities that demean, belittle, and discourage employees as characteristic traits of toxic leadership [4]. As nurses are the largest professional group in healthcare, currently, research on negative leadership behaviours such as abusive supervision within the healthcare sector has predominantly focussed on nurses [5–8].

Recent medical studies have reported various negative consequences of negative leadership behaviours, such as nurse's turnover and silence [5, 9]. Silence is defined as employees intentionally withholding vital information, concerns, suggestions, questions, and opinions about issues related to the job and the organisation [10]. Silence is prevalent in hospitals and has a negative impact on the human resources of healthcare organisations [11]. Previous studies have demonstrated that negative leadership is a significant factor in nurses' silence [10, 12–14]. Leaders' concepts used in the management process (e.g., they think they know more than their subordinates and deny the authenticity of information) can indirectly cause subordinates' silent behaviour [15]. Organizationally, nurses' silence poses a significant threat due to its pervasive detrimental effects, such as the loss of critical information and creative ideas, across all levels of the organization when compared to other passive responses to negative leadership behaviours [16].

Although previous study indicated that nurses' silence not only could have numerous negative impacts on both individuals and organisations but also could impede communication among nurses, obscure patient safety hazards, and hinder the prevention of adverse events individually [9, 16, 17]. There is a lack of systematic reviews on the relationship between negative leadership behaviours and silent behaviours of nurses. In addition, academic studies suggested that negative managerial behaviours of nursing leaders may lead to silencing of nurses, but the relationship is still unclear. This systematic review is conducted to clarify the relationship between negative leadership behaviours and silence among nurses.

## 2. Methods

### 2.1. Design

This systematic review was conducted in accordance with the Preferred Reporting Items for Systematic Reviews and Meta-Analyses (PRISMA) guidelines, and its protocol was registered in PROSPERO with the number CRD42023422847.

### 2.2. Search Methods

A comprehensive search was undertaken by two independent researchers (LZY and WQ) across the following databases: PubMed, Embase, the Cochrane Library, Web of Science, CNKI, Wanfang, and VIP databases, from inception until 30 April 2024, to identify studies that examined the relationship between negative leadership behaviours and nurses' silence. The search strategy involved a combination of relevant MeSH terms and other key terms such as “silence,” “speak out,” “speaking up,” “voice,” “work-related suggestions,” “abusive supervision,” “autocratic leadership,” “negative leadership,” “toxic leadership,” “destructive leadership,” “passive leadership,” and “adverse leadership”. We conducted a manual search of the reference sections of the included publications to identify any articles that may have been overlooked during the initial search. The complete search strategy is outlined in Table 1.

### 2.3. Eligibility Criteria and Study Selection

We included studies with nurses or medical staff (including nurses). Studies assessing negative leadership and silence were included. Abusive supervision and authoritative or toxic leadership were considered negative leadership. The included studies were not limited to publications in languages. Qualitative reports, expert opinions, reviews (literature and systems), meta-analyses, and inaccessible articles are excluded.

First, two researchers (LZY and YYP) identified and removed duplicate records and then sifted through titles and abstracts to remove studies that were not relevant to the research question. Finally, the full-text articles were retrieved and reviewed for inclusion and exclusion, and any discrepancies in the above process were adjudicated by a third researcher (TTH).

### 2.4. Study Selection and Data Extraction

To prevent bias in the data extracted by individuals, two researchers (LZY and YYP) identified and removed duplicate records and then sifted through titles and abstracts to remove studies that were not relevant to the research question. Finally, the full-text articles were retrieved and reviewed for inclusion and exclusion. Any discrepancies in the abovementioned process were adjudicated by a third researcher (TTH) until a consensus was reached.

When appropriate studies were identified, a structured table was designed to obtain the following data from the included studies: authors, publication year, country, study design, hospital setting, sample size, participant characteristics, type of negative leadership, negative leadership measurement tool, silence measurement tool, and results of the correlation between negative leadership and silence among nursing staff.

### 2.5. Quality Appraisal

The selected studies were independently assessed for quality by two researchers (LZY and WQ) of this study. The Newcastle–Ottawa Scale (NOS) was utilised to evaluate the quality of the studies, and any discrepancies were reconciled through consultation with a third researcher (TTH) who received relevant training. The NOS assesses the quality of studies based on the following three domains: selection of study groups, comparability, and assessment of outcomes [18]. Studies that scored ≥7 stars were of high quality [19].

### 2.6. Synthesis of Results

The following three extensive outcomes were considered: (1) negative leadership behaviours, (2) silence, and (3) the relationship between negative leadership behaviours and silence among nurses. The baseline and correlations for the outcome variables were evaluated. A meta-analysis could not be conducted due to the high heterogeneity of the included studies' designs and results reporting.

## 3. Results

### 3.1. Search Results and Quality Appraisal

**Figure 1:**
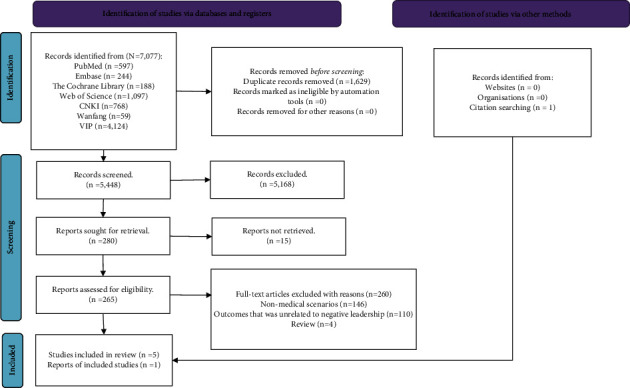
PRISMA flow diagram.

A total of 7,077 studies were initially identified. After removing duplicate articles (*n* = 1,629), 5,448 articles remained for further evaluation regarding their potential inclusion in this systematic review. Of these, 5,168 articles were excluded after screening the titles and abstracts. A final total of 265 articles required full-text review. Following this rigorous process, only six studies were finally included in this systematic review (Figure 1). All six studies were assigned less than seven stars (Table 2).

### 3.2. Characteristics of the Included Studies

The characteristics of the selected studies are summarized in Table 3 [20]. All selected studies used cross-sectional study designs. Four of the included studies [9, 13, 14, 21] were from Asia and two [10, 12] were from Africa. Four of the studies [9, 11, 13, 14] were conducted with nurses and the remaining two [10, 21] were conducted with all medical staff including nurses.

### 3.3. Negative Leadership Behaviours

Table 4 shows that three studies [9, 10, 13] focus on abusive supervision; one [12] on toxic leadership and two [14, 21] on authoritative leadership. The mean scores for abusive supervision were from 1.5 to 2.5 [9, 10, 13]. The mean score of nurses' toxic leadership perception was 3.91 ± 0.51. The mean score of nurses' authoritative leadership perception was 2.97 ± 1.06 [14]. In addition, one study using qualitative interview methods found that authoritative leadership was an important factor affecting the silent behaviour of medical staff [21].

### 3.4. Silence among Nurses

Three studies used identical scales, with mean scores ranging from 3 to 4, indicating that nurses' silence was at a moderate level [13, 14, 21]. In the three remaining studies, the nurses' silence scores were 4.90 ± 0.72, 1.67 ± 0.63, and 2.73 ± 0.77, respectively [9, 10, 12].

### 3.5. Negative Leadership Behaviours and Silence among Nurses

The included articles showed a moderate positive correlation between negative leadership behaviours and nurse' s silent behaviour in four out of six studies, with correlation coefficients of 0.40, 0.49, 0.35, and 0.64, respectively [9, 10, 13, 14]. Li et al. [13] and Zhang [14] also documented that negative leadership behaviour was not only significantly and positively related to nurses' silent behaviour but also significantly and positively related to all three of its dimensions. Qualitative analysis concluded that negative leadership behaviours of hospital managers led to silent behaviours among medical staff [21]. All of the abovementioned studies suggested that negative management behaviours exhibited by hospital leaders exacerbated the silent behaviour of nurses and retained relevant constructs about the organization or manager. However, not all studies have found a positive relationship between negative leadership behaviours and nurses' silence. Farghaly Abdelaliem et al. [12] found a significantly negative correlation between toxic leadership behaviours and nurses' silent behaviour, displaying negative correlation (*r* = −0.77).

Three of the selected articles found that there were other variables that played a role in the relationship between negative leadership behaviours and nurse's silence [9, 10, 14]. Li et al. [9] found that impression management motivation played a partially mediating role in negative leadership behaviours and nurse's silence. Osei et al. [10] showed that proactive personality positively moderated the effect of abusive supervision on medical staff silence. Zhang [14] showed that personality traits partially moderated the relationship between authoritative leadership and nurse's silence.

## 4. Discussion

### 4.1. Clinical Implications

To the best of our knowledge, this is the first systematic review on the relationship between negative leadership behaviours and nurses' silence. Many studies on negative leadership behaviours and employee-silencing behaviours in companies or organizations outside of healthcare have drawn widespread recognition from academia and society, but few are based on healthcare scenarios. Among the six included studies, we found that most studies on negative leadership behaviour were conducted in Asia and Africa, and research was lacking on Western developed countries and regions. This may be related to the different cultural context, with Asian cultures being high power distance cultures, in which subordinates tend to accept and expect to maintain a subordinate relationship with their superiors [22]. A high power distance national culture may, therefore, disincentivize mutual respect and create conditions that are ideal for propagating unchallenged incivility throughout organizations [23, 24]. As for nurses' silence, Asian nurses were more silent, probably because in most Asian cultures, where hierarchies are more rigid and power differentials are greater, speaking up is more challenging than elsewhere and can be risky [25]. Furthermore, many nursing staff chose to remain silent to protect themselves when they perceive problems within their organizations [26].

Four studies [9, 10, 13, 14] showed a significant positive relationship between negative leadership behaviour and silent behaviour among nurses, which is consistent with most studies. Previous studies have showed that authoritative leadership is positively related to employee silence, and the stronger the authoritative leadership style, the more likely it is to lead to silent behaviour [27, 28]. Wang et al. [29] have established that employees' tendency to remain silent is a direct consequence of abusive supervision practices, and this, in turn, influences their attitudes towards work, such as reduced work engagement. Furthermore, another experimental study found that when leaders showed cues that symbolized power, such as direct gaze and voice amplification (intentional or unintentional), subordinates were less vocal and remained silent [30]. Interestingly, for leaders with higher power and authority, the act of raising concerns or suggesting organizational changes can be perceived as a challenge to their management style. Consequently, nurses may choose to remain silent as a means of avoiding direct confrontation or contesting the leader's authority [9]. Moreover, Farghaly Abdelaliem et al. [12] found a significantly negative correlation between negative leadership behaviour and nurse's silence behaviour, reinforcing the previously raised research question of whether the relationship between negative outcomes observed in low-level negative leadership behaviours is appropriate for moderate and high-level negative leadership behaviours [31]. Currently, there is a research gap in the relationship between moderate-to high-level negative leadership behaviours and negative outcomes, and this study fills that gap in the existing literature.

Zhang [14] and Osei et al. [10] found that personality plays a mediating or moderating role in the relationship between negative leadership behaviour and medical staff silent behaviour and that different personalities influence silent behaviour in different ways and with diverse outcomes. Previous research found an association between neurotic personality and silent behaviour [32]. LePine et al. [33] showed that employees' introverted personalities can also impact silent behaviour, and employees with introverted personalities tend to use silence to reduce excessive communication with others. In addition to personality variables, the inclusion of the study showed that impression management motivation could also act as a mediating variable between negative leadership behaviours and nurses' silence [9].

Our findings contribute to the literature in several ways. First, our study extracted and condensed the research on the relationship between negative leadership behaviour and nurses' silence [34] in the medical field. Our findings reveal that negative leadership behaviour would lead nurses to withhold their suggestions and opinions from the organization and keep silent. This was consistent with most previous studies, as well as recent studies on the emergence of negative results such as increased stress [35], increased turnover intention [2], and decreased organizational performance [2, 36] due to leaders' abuse of power. Farghaly Abdelaliem et al. [12] found a negative relationship between negative leadership behaviour and nurse silence behaviour. Zhang et al. [37] also noted the positive impact of negative leadership behaviour in certain situations. Studies have confirmed that negative leadership behaviours do not always lead to negative consequences and can promote employee voice behaviour [38] and creativity [17] to a certain extent. This suggests that the negative behaviour of leaders and the silent behaviour of nursing staff may not just be a simple positive or negative linear relationship, but there may be a curvilinear relationship like a positive and then negative influence. Fleishman et al. [39] pointed out that current research in the field of leadership focuses too much on a simple linear relationship and ignores the nonlinear relationship between the variables. Future research could focus more on whether there is a curvilinear relationship between the two. In addition, most previous studies [10, 12, 40] used nurses' silence as the mediating variable, but this study analysed nurses' silence as the dependent variable to understand the direct impact of negative leadership behaviour on nurses' silence. Second, most previous studies on negative leadership behaviour focused on the enterprise field, and few studies have examined the status quo of negative leadership behaviour and its impact on employee silence based on medical scenarios. The articles included in this study were all medical studies, which broadened the scope of our understanding of negative leadership behaviour. Finally, variables such as various personality traits played different roles in negative leadership behaviour and employee silence, which enriches the research literature on the relationship between negative leadership behaviour and employee silence [34].

### 4.2. Clinical Practice

Sweden, Canada, France, and South Korea have “workplace bullying laws” [40]. Therefore, from the perspective of leadership, hospitals, and other institutions can take various actions such as organizational culture guidance and regular management skills training for head nurses, department directors, and other leaders as a unit to improve leaders' awareness of negative management hazards, strengthen their self-control ability, and minimize the possibility of negative leadership behaviours [41]. Moreover, online and offline reporting centres can be set up in hospitals and other institutions to facilitate nurses or patients to report negative management at anytime and anywhere [40]. Managers in the medical field face greater pressure and challenges than other managers. Therefore, psychological counselling rooms can provide timely counselling when they encounter problems to prevent the occurrence of negative leadership behaviours. Organizations need to be highly attentive towards the psychological and physiological changes in nurses. This necessitates constant communication, understanding of nurse's psychological dynamics, and the establishment of a strong trust relationship. These efforts aim to effectively minimize silent behaviour among nurses. In addition, the implementation of diverse group-building activities can successfully alleviate work-related pressure, enabling nurses to effectively release negative emotions. In ordinary circumstances, fostering a peaceful and amicable atmosphere within the department becomes essential to encourage nurses' freedom of expression [41].

## 5. Limitations

This systematic review has several limitations. First, the number of selected studies was relatively small, which could lead to limited efficacy of our conclusions. Second, only four included studies focused on nursing staff; the bias estimated may be inevitable. Third, the methodological quality of the included studies varied, potentially introducing a risk of bias. Fourth, using quantitative or statistically presented pooled evidence is preferable from a statistical perspective. However, performing a meta-analysis of the selected studies was challenging due to the inconsistent information provided. Fifth, the available limited, very low-quality evidence does not support an association between negative leadership behaviours and nurses' silence. Further studies are necessary to establish this connection. Finally, the lack of studies from developed Western regions in our inclusion criteria necessitates further exploration when extrapolating the study's results.

## 6. Conclusion

For the most part, it can be concluded that a significantly positive relationship exists between negative leadership behaviours and nurses' silence and that leaders' negative management behaviours lead nurses to choose silence for the sake of self-preservation. This phenomenon is detrimental to the physical and mental health of nurses. Hospitals and hospital leaders should increase their awareness of negative leadership and raise alarms or make improvements.

## Figures and Tables

**Table 1:** Search strategy.

Source	Search strategies
PubMed (*N* = 597)	#1 silence [Title/Abstract] OR speaking up [Title/Abstract] OR speak up [Title/Abstract] OR speak out [Title/Abstract] OR speak [Title/Abstract] OR voice [Title/Abstract] OR safety voice [Title/Abstract] OR safety concern [Title/Abstract] OR worry [Title/Abstract] OR work-related suggestions [Title/Abstract] OR work-related opinions [Title/Abstract] *n* = 86297
	#2 abusive supervision OR abusive manager OR passive leadership OR negative leadership OR destructive leadership OR toxic leadership OR dark side leadership OR exploitative leadership OR intrusive leadership OR despotic leadership OR autocratic leadership OR paternalistic leadership OR adverse leadership OR unethical leadership OR nonphysical abuse *n* = 83747
	#3 #1 AND #2 *n* = 597 Search date: 2024/4/30

Embase (*N* = 244)	#1 silence OR speaking up OR speak up OR speak out OR speak OR voice OR safety voice OR safety concern OR worry OR work-related suggestions OR work-related opinions *n* = 188221
	#2 abusive supervision OR abusive manager OR passive leadership OR negative leadership OR destructive leadership OR toxic leadership OR dark side leadership OR exploitative leadership OR intrusive leadership OR despotic leadership OR autocratic leadership OR paternalistic leadership OR adverse leadership OR unethical leadership OR nonphysical abuse *n* = 8663
	#3 #1 AND #2 *n* = 244 Search date: 2024/4/30

Web of Science (I = 1097)	#1 TS = (silence OR speaking up OR speak up OR speak out OR speak OR voice OR safety voice OR safety concern OR worry OR work-related suggestions OR work-related opinions) *n* = 726065
	#2 TS = (abusive supervision OR abusive manager OR passive leadership OR negative leadership OR destructive leadership OR toxic leadership OR dark side leadership OR exploitative leadership OR intrusive leadership OR despotic leadership OR autocratic leadership OR paternalistic leadership OR adverse leadership OR unethical leadership OR nonphysical abuse) *n* = 18403
	#3 #1 AND #2 *n* = 1097 Search date: 2024/4/30

The Cochrane Library (*N* = 188)	#1 silence OR speaking up OR speak up OR speak out OR speak OR voice OR safety voice OR safety concern OR worry OR work-related suggestions OR work-related opinions *n* = 18328
	#2 abusive supervision OR abusive manager OR passive leadership OR negative leadership OR destructive leadership OR toxic leadership OR dark side leadership OR exploitative leadership OR intrusive leadership OR despotic leadership OR autocratic leadership OR paternalistic leadership OR adverse leadership OR unethical leadership OR nonphysical abuse *n* = 950
	#3 #1 AND #2 *n* = 188 Search date: 2024/4/30

CNKI (*N* = 768)	#1 SU = (speaking up + speak up + speaking out + voice + silence + safety voice + safety concern + speak) *n* = 209986
	#2 FT = (abusive supervision + abusive manager + passive leadership + negative leadership + destructive leadership + toxic leadership + dark side leadership + adverse leadership) *n* = 8042
	#3 #1 AND #2 *n* = 768 Search date: 2024/4/30

Wanfang (*N* = 59)	#1 Title/Abstract:(speaking up or speak up or speak out or voice or silence or safety voice or safety concern or speak) *n* = 51306
	#2 all Fields:(abusive supervision or abusive manager or passive leadership or negative leadership or destructive leadership or toxic leadership or dark side leadership or adverse leadership) *n* = 3562
	#3 #1 AND #2 *n* = 59 Search date: 2024/4/30

VIP (*N* = 4124)	#1 *T* = (speaking up OR speak up OR speak out OR voice OR silence OR safety voice OR safety concern OR speak) *n* = 56964
	#2 *T* = (abusive supervision OR abusive manager OR passive leadership OR negative leadership OR destructive leadership OR toxic leadership OR dark side leadership OR adverse leadership) *n* = 154813
	#3 #1 AND #2 *n* = 4124 Search date: 2024/4/30

**Table 2:** Quality assessment of included studies using the Newcastle–Ottawa Scale (NOS).

Author	Year	Selection				Comparability	Exposure			Quality (9 point)
		Is the case definition adequate (1 point)	Representativeness of the cases (1 point)	Selection of controls (1 point)	Definition of controls (1 point)	Comparability of cases and controls on the basis of the design or analysis (2 point)	Ascertainment of exposure (1 point)	Same method of ascertainment for cases and controls (1 point)	Nonresponse rate (1 point)	
Li et al.	2024	1	1	0	0	1	1	1	1	6
Farghaly Abdelaliem et al.	2023	1	1	0	0	1	1	1	1	6
Osei et al.	2022	1	1	0	0	1	1	1	1	6
Pang	2022	1	1	0	0	0	0	1	1	4
Zhang	2021	1	1	0	0	1	1	1	1	6
Li et al.	2014	1	1	0	0	0	1	1	1	5

**Table 3:** Main characteristics of the included studies in the systematic review.

Nos.	Study	Study design	Hospital setting	Sample size	Sex	Age(years)	Education (%)	Professional title	Marital status	Work seniority (years)
1	Li et al., 2024, China	Cross-sectional	A hospital in Zhejiang, China	419	6 males and 413 females	21–24 (25.5%)	Bachelor and above (71.4%) Other (28.6%)	Nurses (31.7%)	Married (56.3%)	<5 (37.7%)
						25–29 (23.9%)		Nurse practitioners (38.9%)	Unmarried (43.2%)	5–9 (21.7%)
						≥30 (50.6%)		Nurse-in-charge or above (29.4%)	Other (0.5%)	≥10 (40.6%)

2	Farghaly Abdelaliem et al., 2023, Egypt	Cross-sectional	A hospital in Alexandria, Egypt	750	163 males and 587 females	<35 (34.9%)	Bachelor and above (95.7%)	NA	Married (52.3%)	<5 (43.5%)
						≥35 (65.1%)	Other (4.3%)		Unmarried (26.0%)	5–10 (56.5%)
									other (21.7%)	

3	Osei et al., 2022, Ghana	Cross-sectional	Five hospitals in Ghana	300	138 males and 162 females	30 ± 0.77	First degree (41.6%)	NA	NA	5 ± 1.28
							Other (58.4%)			

4	Pang, 2022, China	Cross-sectional	A hospital in Henan, China	267	114 males and 153 females	≤25 (10.5%)	Bachelor and above (87.6%)	Primary titles (50.6%)	Married (76.0%)	≤5 (37.1%)
						26–35 (52.8%)	Other (12.4%)	Intermediate titles (33.3%)	Unmarried (23.6%)	6–10 (21.3%)
						>35 (36.7%)		Senior titles (16.1%)	Other (0.4%)	>10 (41.6%)

5	Zhang, 2021, China	Cross-sectional	Five hospitals in Henan, China	560	63 males and 497 females	<25 (25.2%)	Bachelor and above (54.5%)	Primary titles (82.3%)	Married (49.1%)	≤5 (52.0%)
						25–30 (50.7%)	Other (45.5%)	Intermediate titles (15.4%)	Unmarried (49.5%)	6–10 (33.2%)
						>30 (24.1%)		Senior titles (2.3%)	Other (1.4%)	>10 (14.8%)

6	Li et al., 2014, China	Cross-sectional	Five hospitals in Harbin, China	284	13 males and 271 females	28.10 ± 5.97	Bachelor and above (57.7%) Other (42.3%)	Nurses (55.3%) Senior nurses (28.2%) Supervisor nurses (13.7%) Chief nurses or deputy chief nurses (2.8%)	Married (40.1%) Unmarried (58.5%) Other (1.4%)	6.33 ± 6.85

**Table 4:** Negative leadership and silence measures with parameters used in the included studies.

Nos.	First author, year	Negative leadership type	Scales used by negative leadership	Negative leadership Mean ± SD	The scale used in silence	Silence Mean ± SD	Acquiescent silence	Defensive silence	Indifferent silence	Correlation coefficient (silence and negative leadership)
1	Li et al., 2024, China	Abusive supervision	Tepper et al. (2004)	1.31 ± 0.48	Richard et al. (2021)	1.67 ± 0.63	NA	NA	NA	0.40^*∗∗*^
2	Farghaly Abdelaliem et al., 2023, Egypt	Toxic leadership	Zhang et al. (2020)	3.91 ± 0.51	Knoll M et al. (2013)	4.90 ± 0.72	5.52 ± 0.63	NA	NA	−0.77^*∗∗*^
3	Osei et al., 2022, Ghana	Abusive supervision	Tepper et al. (2004)	2.28 ± 0.79	Parker et al. (2009)	2.73 ± 0.77	NA	NA	NA	0.35^*∗∗*^
4	Pang, 2022, China	Authoritative leadership	Interview	NA	Zheng et al. (2008)	3.19	3.73 ± 0.85	3.38 ± 0.95	2.47 ± 0.78	NA
5	Zhang, 2021, China	Authoritative leadership	Zheng et al. (2000)	2.97 ± 1.06	Zheng et al. (2008)	3.53 ± 0.81	3.91 ± 0.85	3.50 ± 1.00	3.17 ± 1.04	0.64^*∗∗*^
6	Li et al., 2014, China	Abusive supervision	Tepper et al. (2004)	2.35 ± 1.50	Zheng et al. (2008)	3.93 ± 1.28	4.33 ± 1.47	4.05 ± 1.53	3.42 ± 1.51	0.49^*∗*^

^*∗*^ *p* < 0.05; ^*∗∗*^*p* < 0.01.

## Data Availability

The data used to support the findings of this study are available from the corresponding author upon reasonable request.
